# A statewide, cross‐sectional evaluation of the knowledge and level of concern of rabies among South Carolina residents

**DOI:** 10.1111/zph.13001

**Published:** 2022-09-30

**Authors:** Lídia Gual‐Gonzalez, Maggie S. J. McCarter, Megan Peebles, Melissa S. Nolan

**Affiliations:** ^1^ Department of Epidemiology and Biostatistics University of South Carolina Columbia South Carolina USA

**Keywords:** cross‐sectional study design, Knowledge Attitudes and Practices, rabies, South Carolina, statewide survey

## Abstract

Animal rabies cases have increased steadily in South Carolina (SC) for the past decade. An understanding of the population's awareness and understanding of the disease is needed to tailor public health interventions. A marketing list‐serv of SC residents' email addresses was used to recruit anonymous respondents for a Knowledge Attitudes and Practices (KAP) electronic survey. A total 516 South Carolinians completed the 31‐question survey. Quantile regression and a Pearson's correlation evaluated potential associations between respondent's rabies knowledge and their attitudes and practices. Knowledge was assessed on topics of rabies biology, state animal case counts and rabies pet‐related laws. Level of concern and level of knowledge were positively correlated. Additionally, statewide hotspot analysis revealed geographic areas warranting targeted public health interventions; counties with low public concern juxtapositioned with high animal rabies case counts. This study demonstrates the utility of statewide KAPs to gauge populations rabies perception and related preventative actions to tailor appropriate educational programs to limit human‐animal rabies exposures.

**Impacts**

- This is the first Statewide Rabies Knowledge, Attitudes and Practices Survey in South Carolina, a high‐risk state with steadily increasing animal rabies case counts over the past decade.
- Geographic high‐risk hotspots across South Carolina were identified for priority public health education given the lack of community rabies concern combined with high animal rabies cases.
- Spearman's correlation revealed that higher Rabies knowledge was associated with greater concern for owned animal health.

## 1 INTRODUCTION

Rabies virus is a neurotropic virus that causes neurologic disease and is considered one of the deadliest zoonoses globally (Brunker & Mollentze, 2018; World Health Organization, 2021). Post‐exposure prophylaxis (PEP) is only effective if administered prior to the onset of symptoms, and its effectiveness decreases the further out treatment is administered from initial exposure (Brunker & Mollentze, 2018; World Health Organization, 2010, 2021). In the United States (US), human rabies cases are mostly imported (Pieracci et al., 2019) and the number of human deaths has been decreasing; the last US‐reported deaths being in 2021 when three people were exposed to bats and died for not seeking PEP (Kunkel et al., 2022). Despite public health accomplishments, sporadic human cases happen and animal rabies cases continue to rise (Ma et al., 2020).

Despite waning concern for rabies in the US, the Center for Disease Control and Prevention (CDC) gathers data collected yearly at state‐level through health departments and national wildlife services and creates surveillance reports (Ma et al., 2020, 2021). South Carolina (SC) tests suspected animal specimens at the state public health laboratory, and the SC Department of Health and Environmental Control (SC DHEC) reports all positive animal cases to the CDC. In 2011, SC reported its most recent human death: a woman who died after close contact with a bat in her home (Rupprecht et al., 2013). This case was paramount as no bite was reported and the patient was unaware of the exposure, delaying medical care until the onset of symptoms. In the decade following this case, there has been a steady increase in the detection of rabies‐positive animals in the state (SC DHEC, 2020). Public awareness regarding this disease continues to be crucial to maintain low risk of exposure and to prevent fatal cases. When designing effective statewide public health interventions, tailored material is often warranted given population‐level demographic and cultural heterogeneity. In this short communication, we present the results of a statewide rabies knowledge, attitudes, and practices survey garnered at elucidating key improvement opportunities for public health practitioners to address when designing future public health interventions.

## 2 MATERIALS AND METHODS

In June 2021, an electronic rabies knowledge, attitudes and practices questionnaire was created using REDCap software (Research Electronic Data Capture) (Harris et al., 2009, 2019). The questionnaire was comprised of 30 questions related to rabies transmission, animal hosts, infected animal behaviours, animal and human rabies epidemiology statewide, treatment basics, suspected case protocols, state‐provided services, animal vaccination laws, one's rabies concerns/beliefs, rabies‐related practices for pet owners and basic demographics (Appendix S1). Email requests were sent to SC residents to complete this questionnaire; addresses were obtained through a proprietary marketing listserv, purchased from Mailers Heaven.

### 2.1 Ethical statement

The University of South Carolina Institutional Review Board determined the study to be exempt from human research due to the anonymous design of the survey.

### 2.2 Statistical analysis

Descriptive analyses were performed to define the sample population and the socio‐demographic characteristics. Pearson's Chi‐square and Fisher's exact tests with Chi‐square Pearson's residuals were performed to evaluate the association between demographics and each question individually. A Questionnaire Knowledge Score (QKS) was created as a summation of correct responses. The maximum score for the QKS was 18 points using questions 2 to 19 from the questionnaire (Appendix S1). A quantile regression model was run to the 50th percentile to explore the association between the QKS and the demographic characteristics. Spearman's correlation was used to evaluate the association between QKS and the level of concern. All statistical analyses were performed using SAS 9.4 and SAS Studio 3.8 (SAS Institute).

### 2.3 Geographic distribution

A False Discovery Rate corrected hotspot analysis of the respondents' concern of their pet getting rabies and a choropleth bivariate map of the knowledge score and total animal positives by county were performed using ArcGIS® Pro Version 2.8.2 (ESRI). Count of rabies‐positive animals per county for the past 10 years was abstracted from the state health department's website publicly available reports (Data available at: https://scdhec.gov/health/diseases‐conditions/insect‐or‐animal‐borne‐disease/rabies/data‐reports‐rabies).

## 3 RESULTS

### 3.1 Respondent characteristics and descriptive analysis

A total of 248,109 emails were sent between June and July 2021 using a brief, scripted invitation letter. UofSC's outlook restricts a daily maximum of 4999 emails sent; therefore, emails were sent from one of three accounts: rabies@sc.edu (a general study account), lidiag@email.sc.edu (graduate research assistant) or msnolan@mailbox.sc.edu (the faculty advisor). Email receipt or flagging of emails as ‘junk’ was unable to be assessed. As of July 26, 2021, 534 surveys were answered, of which 516 were complete and used in the final analysis. Response rates for each question as well as the correct answers are shown in Table 1. The overall response rate was low (0.22%), but not unexpected given the lack of direct incentive for survey completion. Of the 516 survey respondents, 62.7% were female. The majority of respondents were above 45 years old, with 40.1% being 66 years or older, lived in suburban areas (56.6%), 34.4% living in rural and 9.0% in urban areas. Respondents were mostly Caucasian (82.9%) and of high education: 16.5% had some college, 28.2% a college degree and 36.7% a graduate degree.

**TABLE 1:** Questionnaire correct answers' response rates and the statistical analysis for each group

Rabies virus basic biology	(*N* = 516)	Trend^†^	*p* Values for Chi^2^ and Fisher's				
			Gender	Age	Location	Race	Education
			Male^‡^	<56 years old^‡^	Rural^‡^	Non‐white^‡^	Highschool^‡^
How can someone get rabies? Saliva‐infected animal (bite/scratch)	509^a^ (99.4%)	(I)	0.5*	0.06*	0.4*	0.09*	0.2*
Any mammal can get infected and transmit rabies (T).	428^b^ (83.9%)	(I)^§^	0.6^¶^	0.1^¶^	0.5^¶^	0.6^¶^	0.8^¶^
Most animals infected will act differently (wobbly, paralysed, fear of water) (T).	368^a^ (71.9%)	(I)	0.7^¶^	**0.02** ^¶^	0.7^¶^	0.3^¶^	0.05^¶^
Sometimes, wild animals infected may seem friendly/healthy (T).	412^c^ (80.3%)	(I)	0.05^¶^	0.8^¶^	0.6^¶^	**0.009** ^¶^	0.3^¶^
What are the symptoms of rabies in people? Irritability, fever, headache, paralysis and convulsions.	439^d^ (88.3%)	(I)^§^	**0.047** ^¶^	**0.01** ^¶^	0.06^¶^	0.3^¶^	0.9^¶^
People with rabies can survive without treatment (F).	459^e^ (91.8%)	(I)^§^	0.1^¶^	0.6^¶^	0.2^¶^	0.4^¶^	0.9^¶^
People who have been bitten/scratched by an infected animal will need a series of shots over a 2‐week period to stop from developing rabies (T).	477^f^ (95.6%)	(I)^§^	0.4^¶^	0.8^¶^	0.8^¶^	0.6^¶^	0.6^¶^
What are the correct steps to take if someone gets exposed to rabies? Immediately wash bite/wound site with soap and water, contact a doctor.	483^g^(97.4%)	(I)^§^	0.1*	0.2*	0.2*	0.1*	0.06*
Animal vaccines are available for*: Dogs; Cats; Ferrets; Agriculture animals.	30 (5.8%)	(C)^§^	0.02^¶^	0.9^¶^	0.3^¶^	0.3^¶^	0.7^¶^
Situation in South Carolina							
Common carriers of rabies in SC? Raccoons, skunks, foxes and bats.	481^c^ (93.8%)	(I)	0.7^¶^	0.2^¶^	0.4^¶^	0.2^¶^	0.5^¶^
How many animals test positive each year in SC? Between 100–200.	212^h^ (41.5%)	(C)	0.5^¶^	0.5^¶^	0.1^¶^	0.4^¶^	0.9^¶^
How many rabies PEP are given in SC? More than 1000.	32^d^ (6.4%)	(I)^§^	0.06^¶^	0.9^¶^	0.3^¶^	**0.03** ^¶^	0.9^¶^
The number of rabies human deaths in SC each year is: Less than 5.	347^d^ (69.7%)	(I)^§^	0.2^¶^	0.9^¶^	0.3^¶^	**0.01** ^¶^	0.4^¶^
SC law mandates all animal bites must be reported to local DHEC environmental affairs office (T).	407^i^ (82.4%)	(I)	**<0.001** ^¶^	**0.03** ^¶^	0.4^¶^	0.7^¶^	0.4^¶^
In SC, pet owners are required by law to get their pet vaccinated against rabies (T).	441^i^ (89.3%)	(I)	0.3^¶^	**0.04** ^¶^	0.1^¶^	**0.02** ^¶^	0.1^¶^
Where can you take your pet to get vaccinated?* Humane society or rescue shelter; Mobile vaccination site; One's veterinarian.	170 (33.0%)	(I)	0.8^¶^	0.05^¶^	0.2^¶^	**0.009** ^¶^	0.2^¶^
Rabies cases only occur in same two counties each year in SC and is not a concern in any other counties (F).	494^g^ (99.6%)	(I)	0.6*	0.5*	0.4**	0.3**	0.2**
In the past 5 years in SC, the number of animal rabies cases have: Increased.	169^j^ (34.1%)	(C)^§^	0.2^¶^	0.4^¶^	0.3^¶^	0.4^¶^	**0.02** ^¶^

* *Note*: Bold emphasize the statistically significant findings (*p* < 0.05).

* Respondents had multiple answer choices, percentages of the right answer are calculated by adding all the correct answers. Missing values: a = 4, b = 6 c = 3 d = 19 e = 16 f = 17 g = 20 h = 5 i = 22 j = 21.

^†^ Distribution trends on each analysis were obtained from Pearson's Residuals, I: for trends to incorrect answers, C for trends to correct answers.

^‡^ Group with the greatest trend. The analysis compared 2 groups for gender, age, location and race: males vs. females, elderly vs. <56 years old, urban vs. rural, white vs. non‐white. There were three groups compared for education: high school or less, associates degree or some college, college degree or more.

^§^ Some college was the group of greatest trend.

^¶^ Pearson's chi‐square test.

** Fisher's Exact test.

### 3.2 Respondents knowledge, pet‐related rabies practices and exposures

The median knowledge score was 13.0 (95% CI: 12.0–15.0) out of 18.0, and the median per cent of correct answers was 72.2% (95% CI: 66.7–83.3). No statistically significant differences were found between demographic groups for the median QKS knowledge score. However, several individual questions were statistically significant by demographic group (Table 1). The majority of respondents were accurate on rabies biology knowledge questions; however, only 6% were aware that animal vaccines are available for dogs, cats, ferrets and agriculture animals. However, most respondents with pets (*n* = 456) reported having no reasons to choose not to vaccinate (93.2%), and 391 (85.7%) reported vaccinating their pets. While 89.3% of pet‐owning respondents correctly responded that state law requires annual vaccination, 33.0% could correctly name a location to get their pet vaccinated.

Respondent awareness was low for the annual animal rabies cases (41.5%), the number of PEP regimens given annually (6.4%), and the increasing epidemiologic trend of rabid animals (34.1%). Encounters were reported by 6.6% of respondents and 4.1% of respondents reported ever being bitten by a wild animal, and 2.1% reported having had a possible exposure with a wild animal in their homes. Further, 10.1% reported having had a close family member or friend treated for rabies exposure. History of personal or close contact with rabid animal encounters was not significantly associated (*p* < .448) with higher QKS scores.

### 3.3 Respondents concern

Regarding the concern about rabies in the community, 33.0% of the respondents reported not being at all concerned, while 67.0% reported some level of concern. Among pet owners (*n* = 456), 54.7% reported not being concerned about their pet contracting rabies while 45.3% reported some level of concern. When being asked about their concern about someone in their family contracting rabies, 53.0% reported not being concerned at all, while 47.0% reported some level of concern.

### 3.4 Spearman's correlation

**FIGURE 1:**
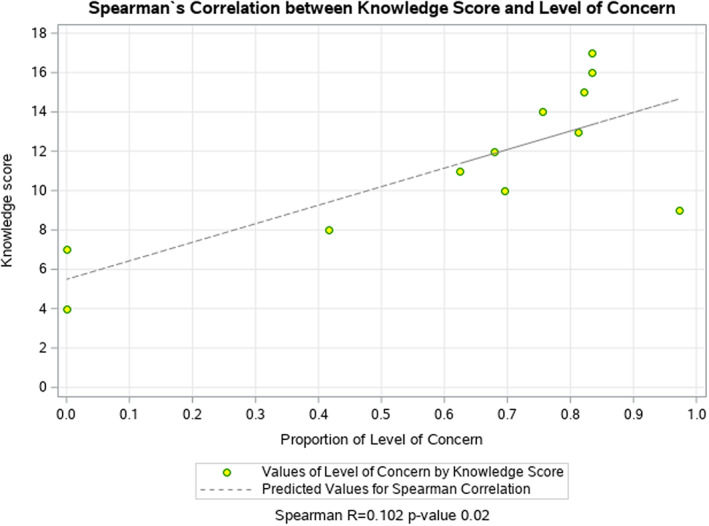
Spearman's correlation demonstrates correlation between respondent rabies knowledge and level of concern about rabies in community. Spearman Correlation between level of concern of getting rabies in the community and the Questionnaire Knowledge Score. The *X*‐axis represents the values for the proportion of level of concern for each knowledge score value. Missing values = 22, *p*‐value = .02.

No significant association was found between demographics and median knowledge score, QKS. However, we found a significant association between demographics (gender, age, race and education level), and the knowledge for several individual questions represented in Table 1. We found a positive correlation between the level of concern among rabies spread in the community and the knowledge score shown in Figure 1.

### 3.5 Geospatial analysis

**FIGURE 2:**
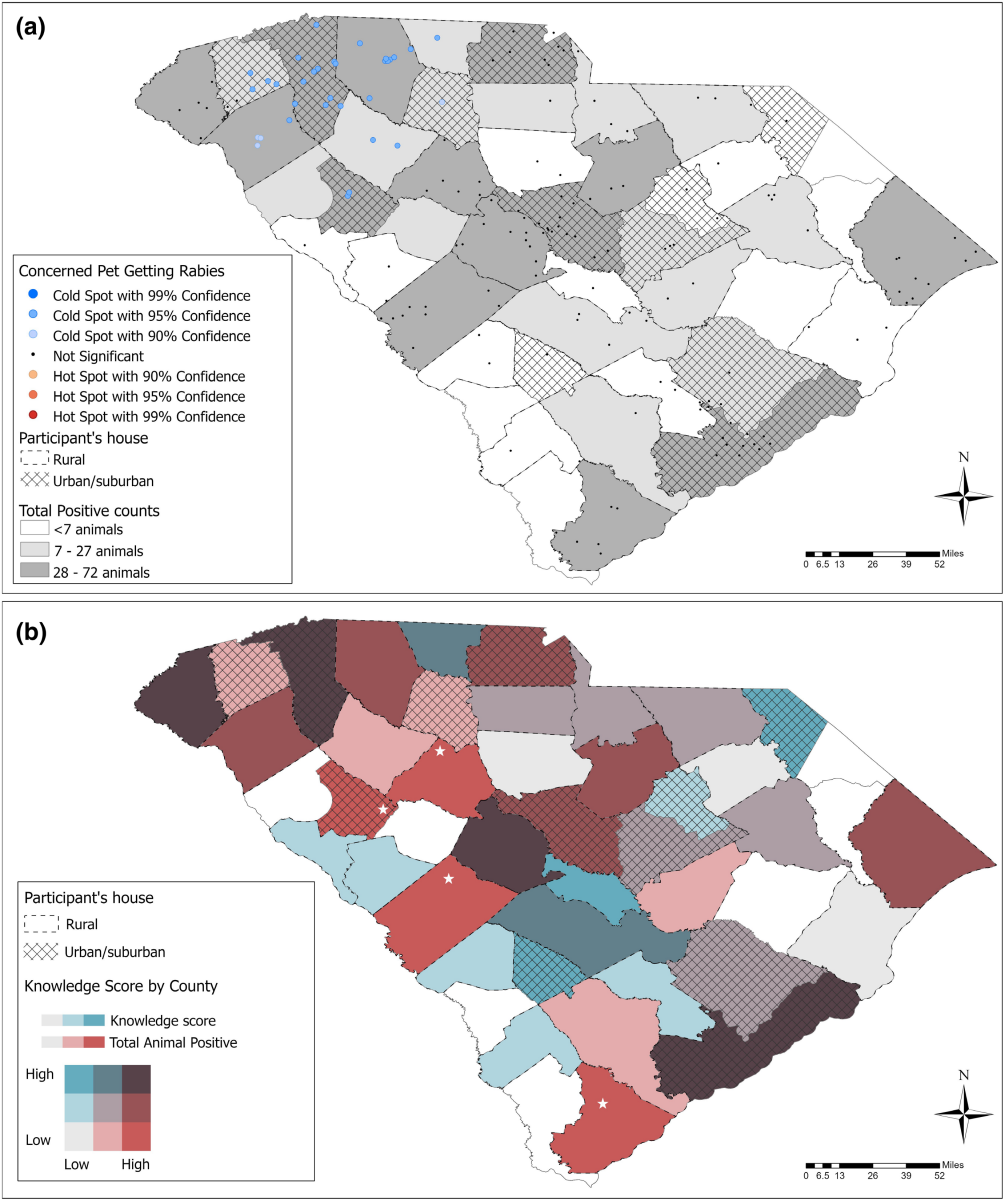
Hotspot analysis revealed key geographic locations exhibiting greater potential transmission risk given high animal rabies positives yet low community concern or low rabies knowledge, by county. (a) A hotspot analysis with statistically significant values representing the low level of concern as a significant spot (blue) and grey scale for the total animal positives by county. (b) Representation of the median Questionnaire Knowledge Score by county (blue gradient) in comparison with the total number of positive animal cases in the past 10 years by county (maroon gradient). The maroon color with white stars represent areas of interest for targeted intervention with high total animal positives and low knowledge score. Counties with more participants living in urban/suburban areas are shown in crosshatch in both maps.

No statistically significant clusters were found in the hotspot analysis for the knowledge score by county. However, a hotspot analysis for the level of concern found statistically significant cold spots of participants' level of concern about their pet getting rabies (Figure 2a). A constructed bivariate map shows the distribution by county of the knowledge score and the total number of positive animal cases from the last 10 years (Figure 2b).

## 4 DISCUSSION

Animal rabies case count has recently increased in SC, with more than 1,000 residents requiring rabies PEP annually (SC DHEC, 2022). The current study demonstrates the utility of a statewide survey to assess key determinants for enhancing future public health education and outreach interventions. South Carolina respondents demonstrated great familiarity with basic rabies knowledge (transmission route, symptoms of disease, etc.); however, they were largely unaware of the epidemiology and the gravity of the animal rabies problem statewide. Notably, geospatial analysis identified key at‐risk communities that exhibited low rabies knowledge yet high animal rabies case count. Specifically, we found those respondents in rural communities that seem significantly less concerned of their pets contracting rabies, despite the increase in animal cases, bringing to light a need for more targeted surveillance and interventions in the rural communities in the upstate region in Figure 2 (Ma et al., 2020, 2021). Some counties were considered areas of potential vulnerability (white star).

Although animal cases have declined in the United States overall, animal rabies cases have steadily increased in SC since 2011 (Rupprecht et al., 2013; SC DHEC, 2020): including a 48% increase between 2018 and 2019 alone (Ma et al., 2021). Evidence suggests rabies is a potentially reemerging, and often neglected, public health concern nationally and education is crucial to avoid fatalities (Kunkel et al., 2022; Pieracci et al., 2019; Whitehouse et al., 2021). Unlike other states, rabies management programs in SC do not include treatment for wildlife, such as using oral vaccination in baits (Davis et al., 2019; Ma et al., 2021). An important limiting factor for oral vaccination bait use is the inability for private vendors to purchase from the USDA, the current licencing holder. Additionally, there are no active rabies surveillance initiatives, although testing is available to track possible exposures (Animals, Livestock and Poultry, 2002). Consequentially, residents of SC are at increased risk of contact with infected wildlife (DHEC Media Relations, 2021), therefore, indicating urgency for public health action.

This study is not without limitations. Most notably, a poor response rate and subsequent small sample size potentially influenced analytic power and/or response bias. The authors hypothesize that a considerable number of emails were flagged as ‘junk’ and the veritable response rate was likely higher among those who actually received the survey invitation in their primary inbox. A marketing listserv may not be a useful strategy for recruitment of participants, and a greater sample could have been obtained if using alternative contact method such as phone calls or mailing invitation letters, as well as providing a small compensation for the participation. Future studies with larger sample sizes would benefit from further analysis.

As noted in Figure 2b, urban/suburban areas generally had greater knowledge and animal counts, whereas several rural areas had low rabies knowledge yet high rabies‐positive animal counts. This finding should be assessed with caution, as animal testing is likely more frequent in urban areas, potentially due to a greater population size that can observe and report suspected rabid animals. Further, urban/suburban counties have a potentially greater tax revenue base to fund local animal control efforts. In support of our findings; however, a recent article on North American rabies control highlights that the greatest risk for human rabies exists in the geographical areas where sylvatic and domestic animals interface allowing for greater spillover events (Fehlner‐Gardiner, 2018). While suburban areas with new housing developments are a likely area for this type of interaction, rural homesteads with fragmented habitation are additional sources of sylvatic‐domestic rabies spillover event risk (Velasco‐Villa et al., 2017). To obtain prevalence estimates that are comparable across the state, consistent sampling is warranted. Although we could not account for the number of total tests performed, this analysis aims to bring up the need for an active surveillance program for rabies in SC to address that question.

Additionally, our sample was relatively homogeneous; participants were mostly Caucasian, more educated and older than the general SC population (Schuler & Deichert, 2019). The self‐selection bias of the respondent group potentially influenced the high rabies knowledge captured in our survey, and future studies should focus on incorporating a more representative survey population. Although the results presented here are not generalizable to the entire population in the state due to the demographic distribution, it is important to acknowledge there is a need for a consistent surveillance across the state to obtain better estimates of the rabies situation in SC.

This short communication aims to highlight awareness and education on rabies are pertinent to its control. Quality education on the nature and risks of rabies could contribute to a more timely potential exposure‐response: a life‐saver (Fooks & Jackson, 2013). Because of this, rabies must not be neglected in the minds of health professionals; this study contributes to providing invaluable information on effectively tailoring public health education initiatives to combat this often‐overlooked disease.

## CONFLICT OF INTEREST

The author(s) declared no potential conflicts of interest with respect to the research, authorship and/or publication of this article.

## Supporting information


Appendix S1

## Data Availability

The data that support the findings of this study are available from the corresponding author upon reasonable request.
