# Supplementary material for: A statewide, cross‐sectional evaluation of the knowledge and level of concern of rabies among South Carolina residents
Source: Zoonoses Public Health. 2022 Sep 30;70(1):103–10. doi: 10.1111/zph.13001 (PMC10092321; doi:10.1111/zph.13001)
Supplement: Supplementary file 1 — Appendix S1 [file ZPH-70-103-s001.docx]

**Supplemental Material 1: South Carolina Rabies Knowledge, Attitudes and Practices Survey, 2021**

1. Have you ever heard of a disease called rabies?
   1. Yes
   2. No
2. Which of the following is correct about how someone can get rabies:
   1. Rabies is transmitted by the bite of a mosquito
   2. Rabies is transmitted by aerosolized droplets or breathing from an infected person
   3. Rabies is transmitted by saliva of an infected animal (for example, a bite or scratch) [correct answer]
3. True or false: Any mammal has the ability to be infected with and transmit rabies:
   1. True [correct answer]
   2. False
4. In South Carolina, which are the most common carriers of rabies?
   1. Raccoons, skunks, foxes, and bats [correct answer]
   2. Bats, humans, and dogs
   3. Dogs, cats and ferrets
   4. Cows, pigs, and goats
5. True or False: Animals infected with rabies will normally be acting differently (for example, appear drunk or wobbly, appear partially paralyzed, have a fear of water).
   1. True [correct answer]
   2. False
6. True or False: Sometimes, wild animals infected with rabies may seem friendly or healthy.
   1. True [correct answer]
   2. False
7. In South Carolina, how many animals test positive for rabies each year?
   1. Less than 100 animals
   2. Between 100 and 200 animals [correct answer]
   3. More than 200 animals

------------------------------------------------------------------------------------------------------(page break)

1. What are the symptoms of rabies in people?
   1. Flu-like illness with cough and nasal congestion
   2. Muscle soreness, feeling tired and foot pain
   3. Irritability, fever, headache, paralysis and convulsions [correct answer]
2. True or False: People with rabies can survive without any medical treatment:
   1. True
   2. False [correct answer]
3. True or False: People who have been bitten or scratched by an infected animal will need to get a series of shots from a doctor over a two-week period to stop the virus from infecting their body.
   1. True [correct answer]
   2. False
4. How many rabies post-exposure treatments are given in South Carolina each year:
   1. Less than 100
   2. Between 100 to 1,000
   3. More than 1,000 [correct answer]
5. The number of rabies human deaths in South Carolina each year is:
   1. Less than 5 [correct answer]
   2. 5 to 50 per year
   3. 100 or more per year

------------------------------------------------------------------------------------------------------(page break)

1. What are the correct steps you should take if you or a household member was exposed to a rabid animal?
   1. Immediately wash the bite/wound site with soap and water, and contact a medical doctor. [correct answer]
   2. Only call a medical doctor if the biting animal appeared rapid or disoriented.
   3. Wait two weeks to see if you develop any symptoms.
2. True or False: South Carolina law mandates that all animal bites must be reported to a local DHEC environmental affairs office.
   1. True [correct answer]
   2. False
3. True or False: In South Carolina, pet owners are required by law to get their pet vaccinated for rabies annually or as directed by a veterinarian.
   1. True [correct answer]
   2. False
4. Animal rabies vaccines are available for: *Check all that apply*
   1. Dogs [correct answer]
   2. Cats [correct answer]
   3. Ferrets [correct answer]
   4. Exotic animals (lynx, civet cats, etc)
   5. Agriculture animals (horses, cattle, sheep, etc) [correct answer]
   6. Circus animals (elephants, tigers, lions, etc)
5. Where can you take your pet to get vaccinated? *Check all that apply*
   1. DHEC annual mass vaccination events [correct answer]
   2. Humane society or rescue shelter [correct answer]
   3. Mobile vaccination site [correct answer]
   4. One’s veterinarian [correct answer]
   5. Other location:
6. True or false: Rabies cases only occur in the same two counties each year in South Carolina, and is not a concern in any other county.
   1. True
   2. False [correct answer]
   3. I do not know
7. In the past ten years in South Carolina, the number of animal rabies cases have:
   1. Decreased
   2. Not changed
   3. Increased [correct answer]
   4. I do not know

------------------------------------------------------------------------------------------------------(page break)

1. How concerned are you about rabies in your community?
   1. Not at all concerned
   2. Somewhat concerned
   3. Concerned
   4. Very concerned
2. How concerned are you about your pet getting rabies?
3. Not at all concerned
4. Somewhat concerned
5. Concerned
6. Very concerned
7. I do not have a pet
8. How concerned are you about someone in your family or household getting rabies?
9. Not at all concerned
10. Somewhat concerned
11. Concerned
12. Very concerned

------------------------------------------------------------------------------------------------------(page break)

1. Do you currently or ever had pets?
   1. Yes
      1. Did any of the following reasons keep you from vaccinating your pet(s)?
         1. Vaccine was too expensive
         2. I do not know where to get a vaccine for my pet
         3. I do not think vaccinations are necessary
         4. Other reason
         5. None of these apply to me
   2. No
2. Do any of the following apply to you: *Check all that apply*
   1. I have reported a rabid animal to authorities
   2. I have vaccinated a pet(s) for rabies
   3. I have been bitten by a wild animal
   4. I have had a possible exposure with a bat or a wild animal in my house
   5. I have had a family member or close friend treated for a rabies exposure
      1. Please describe any incident above:

------------------------------------------------------------------------------------------------------(page break)

***We’re almost done! Please answer these last few questions!***

1. Would you say your house/property is:
2. Urban
3. Suburban
4. Rural
5. What is your home zipcode? _ _ _ _ _
6. What gender do you identify as? *Please choose one*
   1. Male
   2. Female
   3. Non-binary
   4. Transgender
   5. Other
7. What is your age range?.
   1. 18-25 years old
   2. 26-35 years old
   3. 36-45 years old
   4. 46-55 years old
   5. 56-65 years old
   6. ≥65 years old
8. What is your race/ethnicity? *Check all that apply*
9. White
10. Hispanic or Latinx
11. Black or African American
12. Native American or American Indian
13. Asian/Pacific Islander
14. Mixed race
15. Other
16. What is your highest educational degree?
    1. Less than high school
    2. High school graduate or equivalent
    3. Some college
    4. Associates degree
    5. College degree
    6. Graduate school or professional degree
    7. Other

------------------------------------------------------------------------------------------------------(page break)

1. Is there anything else you would like to tell us?

Thank you for your time! For more information on rabies, please visit: Thank you for your time! For more information on rabies, please visit: <https://scdhec.gov/health/diseases-conditions/insect-or-animal-borne-disease/rabies>
